# Supplementary material for: Genetic dissection identifies Necdin as a driver gene in a mouse model of paternal 15q duplications
Source: Nat Commun. 2021 Jul 1;12:4056. doi: 10.1038/s41467-021-24359-3 (PMC8249516; doi:10.1038/s41467-021-24359-3)
Supplement: Supplementary file 1 — Supplementary Information [file 41467_2021_24359_MOESM1_ESM.pdf]

Supplementary Information

Genetic dissection identifies Necdin as a driver gene in a mouse model of paternal 15q duplications

Tamada et al.

Supplementary Information contains

Supplementary Figures 1-9

Supplementary Table 1

Supplementary Figure 1

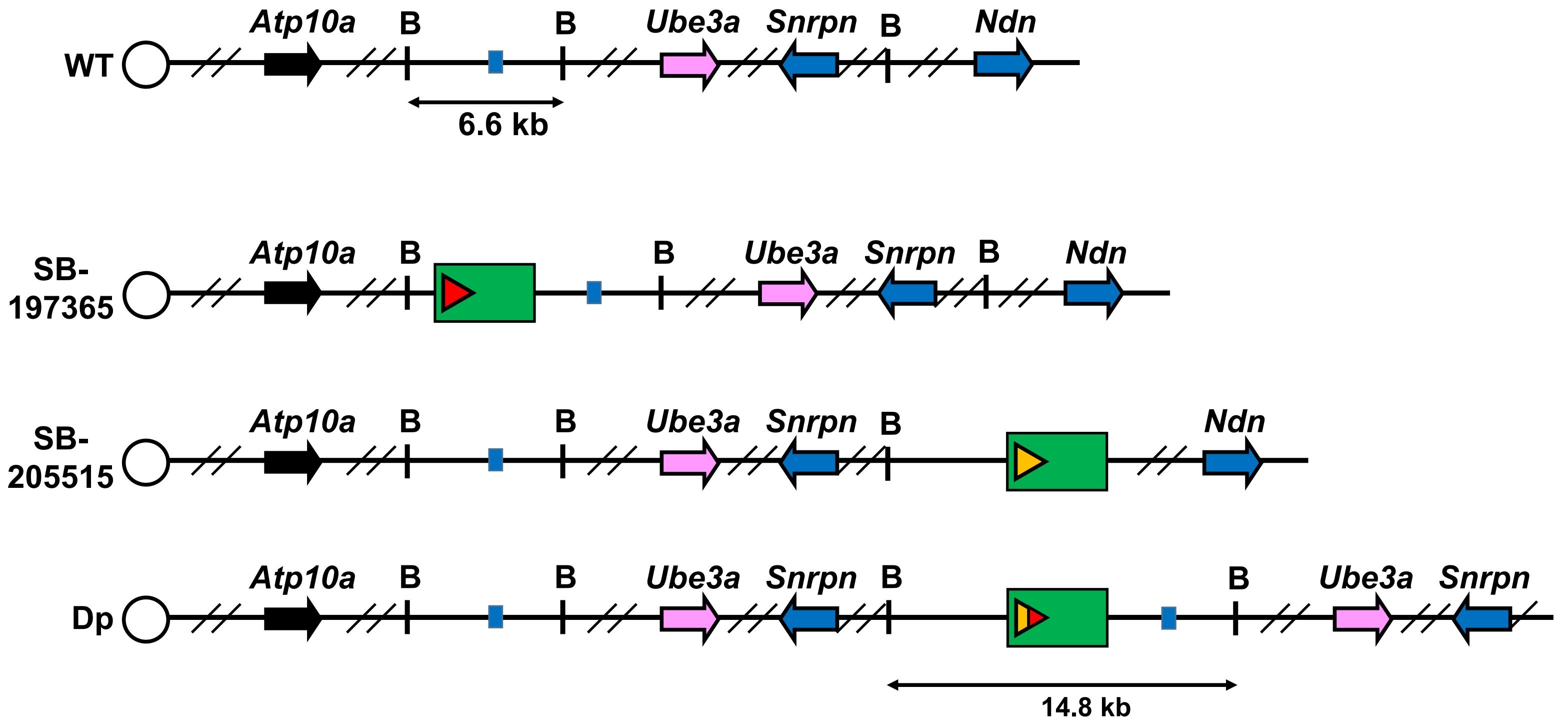

Supplementary Figure 1. Genomic map of 1.5 Mb duplication mice

Genomic configuration and restriction map of 1.5 Mb duplication mice in mouse chromosome 7B-C. Mice with a transposon including a loxP sequence insertions between *Atp10a* and *Ube3a* (SB-197365) and between *Snrpn* and *Ndn* (SB-205515) were obtained from the TRACER database[59]. Duplication of the intervening segment (Dp) was obtained by *in vivo* Cre-mediated recombineering *in trans* with an *Hprt-Cre* transgene[21,22]. Blue box, the probe for Southern blot; B, BsrGI; Green box: transposon; Red and orange triangle; loxP sequence.

Supplementary Figure 2

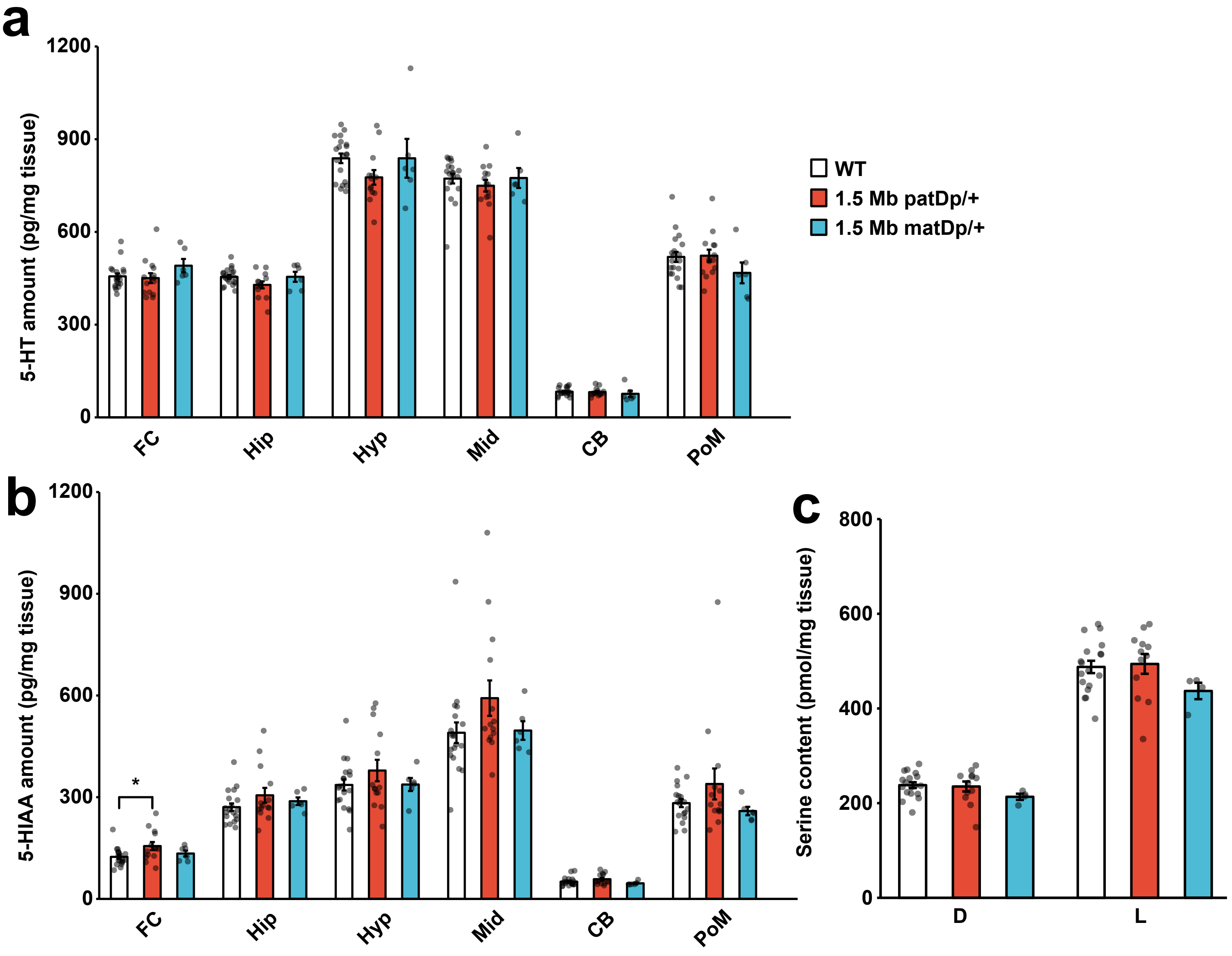

Supplementary Figure 2. Duplication of 1.5 Mb does not affect serotonin and serine amount in the brain
(a-b) Quantification of serotonin (5-HT) and its metabolite 5-hydroxyindoleacetic acid (5-HIAA) in 6 brain regions using HPLC. FC: frontal cortex, Hip: hippocampus, Hyp: hypothalamus, Mid: midbrain, CB: cerebellum, PoM: pons and medulla. N = 19 (WT; 5-HIAA; all brain regions, 5-HT; FC, Mid, CB, PoM), 20 (WT; 5-HT; Hip, Hyp), 13 (*1.5 Mb patDp/+*; 5-HT; Hip, Hyp), 14 (*1.5 Mb patDp/+*; 5-HIAA; all brain regions, 5-HT; FC, Mid, CB, PoM), 6 (*1.5 Mb matDp/+*) biologically independent mice. 5-HIAA : WT vs. *1.5 Mb patDp/+* : $p = 0.028$. *$p<0.05$ (Tukey-Kramer test). **(c)** Quantification of D- and L- serine in frontal cortices. N=18, 12, 4 biologically independent mice for WT, *1.5 Mb patDp/+* and *matDp/+*, respectively. Data are represented as mean +/- s.e.m.

Supplementary Figure 3

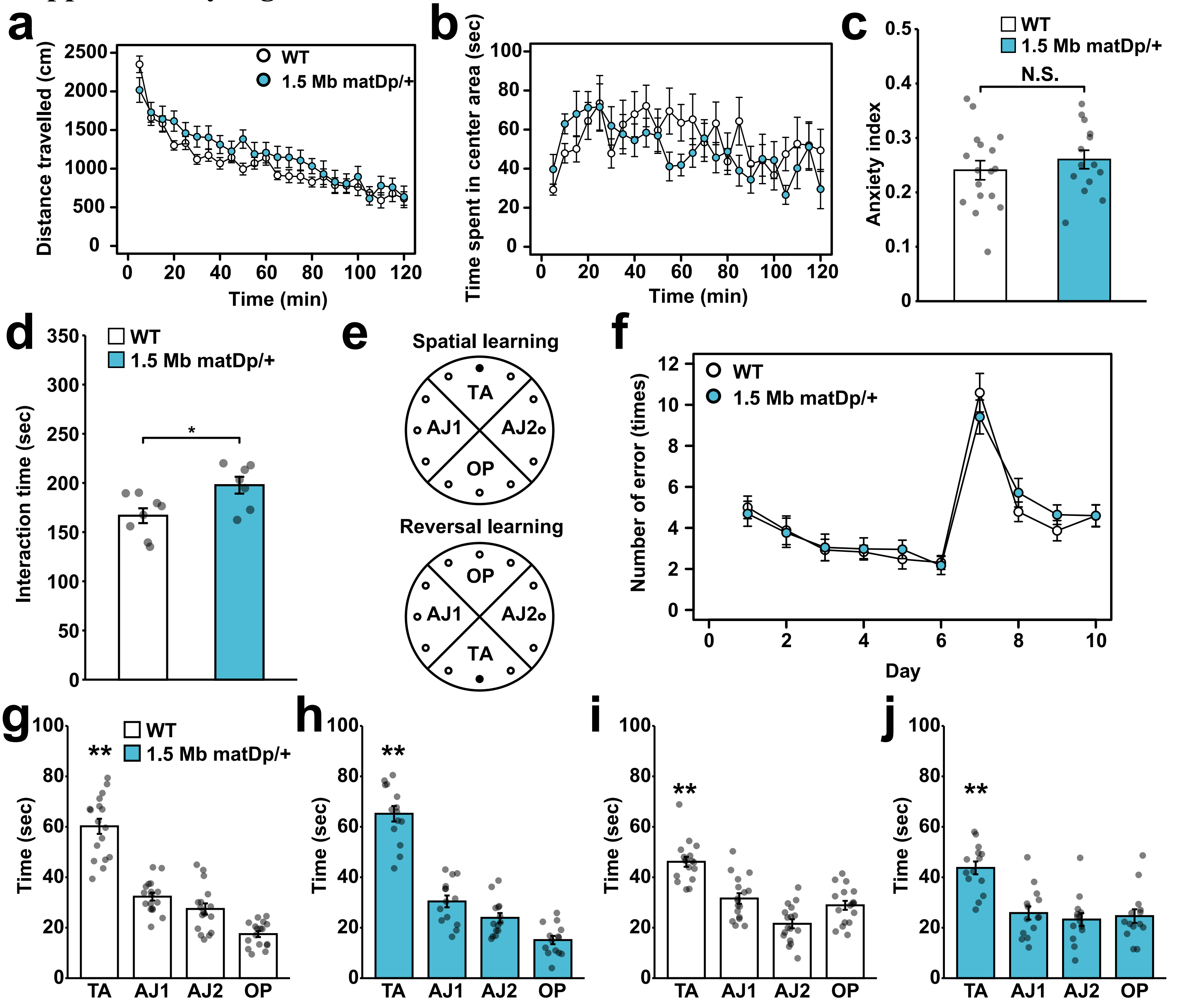

Supplementary Figure 3. Maternal duplication of 1.5 Mb does not induce ASD-like behaviors

(a-c) Open field test in 1.5 Mb matDp/+ mice. Mean values in 5-min segments are plotted including **(a)** locomotor activity and **(b)** time spent in the center area. **(c)** Anxiety index is calculated as distance in the center area divided by total distance during the first 30 min. N=17 (WT) and 14 (*1.5 Mb matDp/+*) biologically independent mice. **(d)** Reciprocal social interaction test. The interaction time for each pair of mice is shown. N=8 (WT), 7 (*1.5 Mb matDp/+*) pairs. WT vs. *1.5 Mb matDp/+* : p = 0.018. ** p < 0.05 (t-test). **(e-j)** Spatial and reversal learning memory was evaluated by the Barnes maze test. **(e)** A schematic view of 2 learning paradigms. TA: target quadrant, AJ1/2: adjacent quadrant and OP: opposite quadrant. A black filled circle indicates the correct hole with an escapable box under the hole. **(f)** The number of errors until reaching the correct target is shown. Spatial and reversal learning was conducted on days 1 to 6 and 7 to 10, respectively. **(g, h)** Results of the probe test on day 6 (**g**: WT, **h**: *1.5 Mb matDp/+*). WT : TA vs. AJ1/AJ2/OP : p < 0.0001, *1.5 Mb matDp/+* : TA vs. AJ1/AJ2/OP: p < 0.0001. **p < 0.01 (Dunnett's test). **(i, j)** Results of the reversal probe test on day 10. N=17 (WT) and 14 (*1.5 Mb matDp/+*) biologically independent mice. WT : TA vs. AJ1/AJ2/OP : p < 0.0001, *1.5 Mb matDp/+* : TA vs. AJ1/AJ2/OP: p < 0.0001. **p < 0.01 (Dunnett's test). Data are represented as mean +/- s.e.m. N.S.: not statistically significant.

Supplementary Figure 4

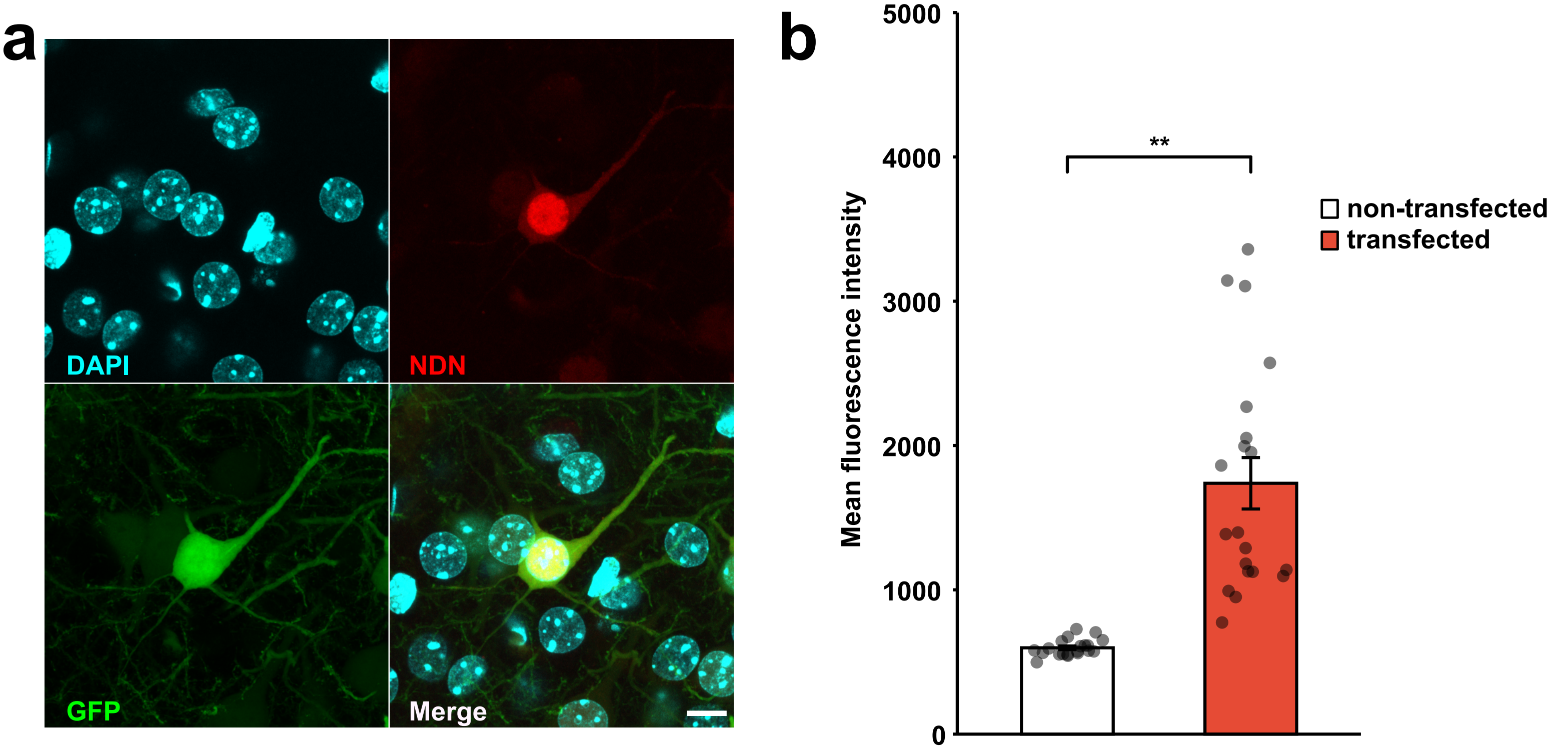

Supplementary Figure 4. Quantification of the expression level of *Ndn* in transfected neurons by *in utero* electroporation

(a) Representative images of *Ndn* transfected cortical neurons. *Ndn* expressing plasmid and GFP plasmid were transfected to cortical neurons by *in utero* electroporation on E15.5. Immunohistochemistry by using NDN antibody was performed on postnatal day 3 weeks old. An arrow and arrowhead indicate transfected and non-transfected neurons, respectively. **(b)** Mean fluorescence intensity per pixel of NDN signal in each nucleus was measured in non-transfected and transfected neurons (N= 20 neurons from 2 biologically independen mice for each group). Non-transfected vs. transfected: $p < 0.0001$ **$p < 0.01$ (t-test). Data are represented as mean +/- s.e.m. Scale bar in **a**, 10 μm.

Supplementary Figue 5

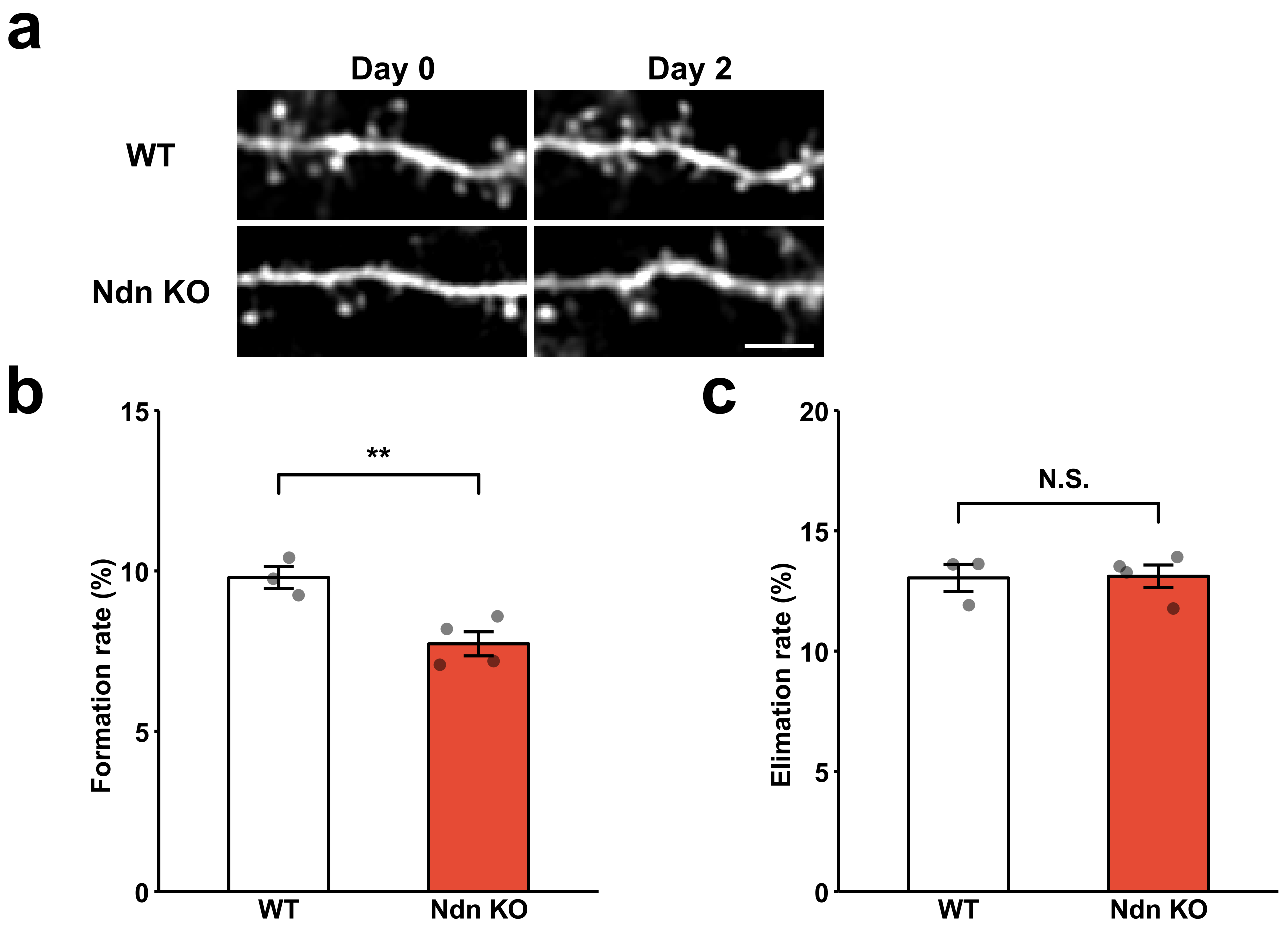

Supplementary Figure 5. *Ndn* KO mice exhibit decreased spine formation rate

(a) Representative images of dendrites in WT and *Ndn* KO mice. **(b)** Spine formation rate and **(c)** elimination rate calculated from WT and *Ndn* KO mice, visualized by *in utero* electroporation of EGFP plasmid. Arrows and arrowheads indicate newly formed and eliminated spines, respectively. The scale bar is 5 μm. N = 3 (WT) and 4 (*Ndn* KO) biologically independent mice. Formation rate : WT vs. *Ndn* KO : p = 0.0099, Elimination rate : p = 0.929. **p < 0.01 (t-test). Data are represented as mean +/- s.e.m. N.S.: not statistically significant.

Supplementary Figue 6

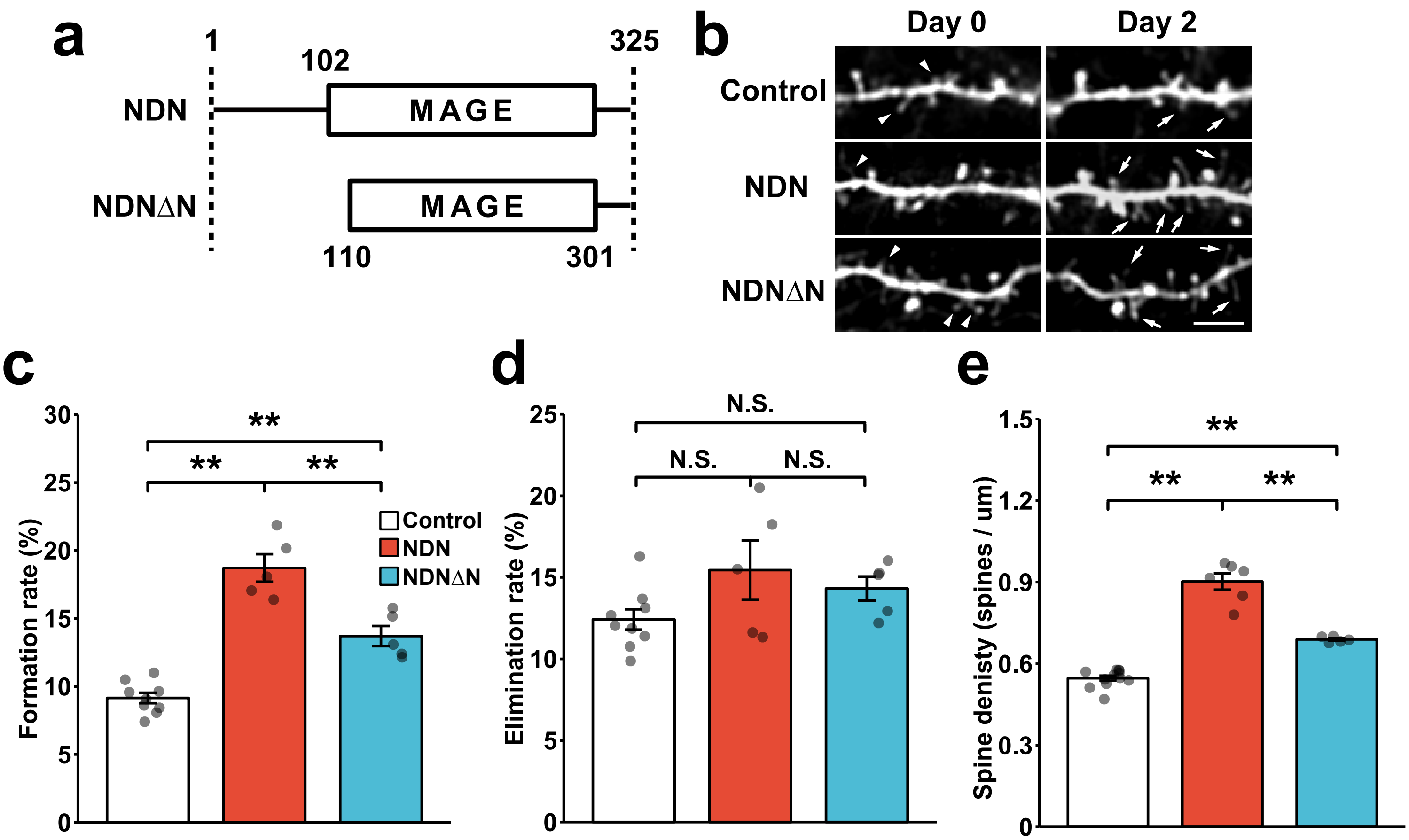

Supplementary Figure 6. N-terminal of NDN partially contributes to dendritic spine formation

(a) A schematic protein structure of intact NDN (325 amino acids) and NDNΔN (110-325). This NDNΔN is reported to lack binding affinities and changes transcriptional activities. MAGE domain was identified using the database of protein domains, PROSITE (http://prosite.expasy.org/). **(b)** Representative images of dendritic spines electroporated with each construct of control, NDN, and NDNΔN. Arrows and arrowheads indicate newly formed and eliminated spines within 2 days, respectively. **(c)** Spine formation rate, **(d)** elimination rate, and **(e)** density with overexpression of each plasmid are shown. Data of control and NDN are the same as in Figure 3c-e. Formation rate : Control vs. NDN : $p < 0.0001$, Control vs. NDNΔN : $p = 0.0003$, NDN vs. NDNΔN : $p = 0.0005$, Elimination rate : Control vs. NDN : $p = 0.115$, Control vs. NDNΔN : $p = 0.398$, NDN vs. NDNΔN : $p = 0.765$. Density: Control vs. NDN : $p < 0.0001$, Control vs. NDNΔN : $p < 0.0001$, NDN vs. NDNΔN : $p < 0.0001$. Arrows and arrowheads indicate newly formed and eliminated spines, respectively. A scale bar indicates 5 µm. (c-e), N = 4 (control) and 5 (NDN and NDNΔN) biologically independent mice. **$p < 0.01$ (Tukey-Kramer test). Data are represented as mean +/- s.e.m. N.S.: not statistically significant.

Supplementary Figue 7

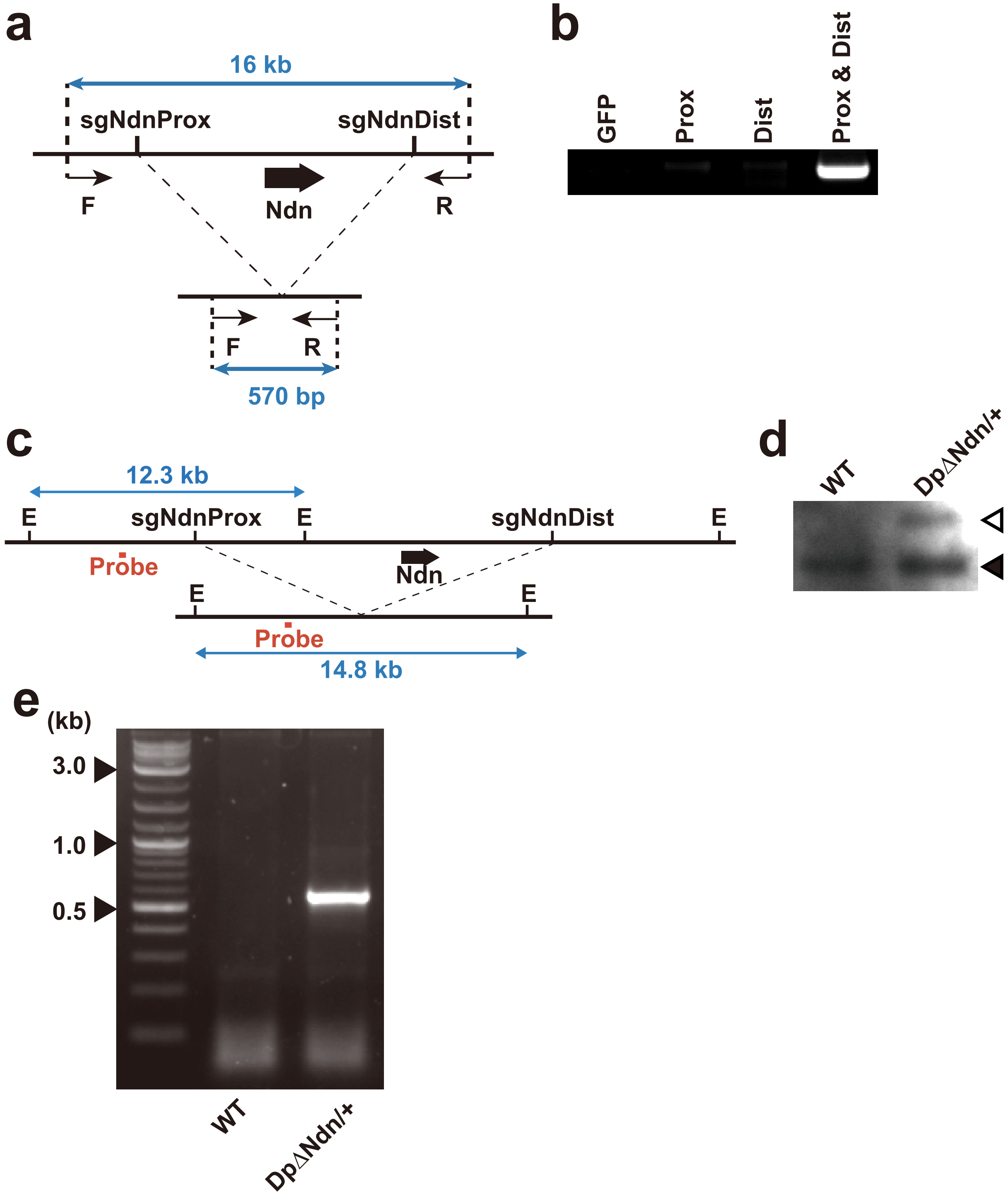

Supplementary Figure 7. Validation of guide RNA for *15q dup*Δ*Ndn* and evaluation of genomic deletion
(a) A schema of deletion detection PCR. Two guide RNAs (sgNdnProx, sgNdnDist) were designed adjacent to the *Ndn* gene. F and R indicate PCR primers used for evaluating genomic deletion. **(b)** Genomic PCR of Neuro2a transfected with guide RNA expressing vectors. **(c)** Design of Southern blot probe. E: EcoRI site. Upper: WT and lower deleted alleles. **(d)** Southern blot using mouse tail genome. White triangle: deletion specific band (14.8 kb) and black triangle: WT band (12.3 kb). Similar results were obtained in 3 biologically independent mice (Source Data). **(e)** Genomic PCR using the tail genome of WT and *15q dup*Δ*Ndn* mice. Similar results were obtained in more than 3 biologically independent mice. The primer pair shown in **(a)** was used for PCR.

Supplementary Figue 8

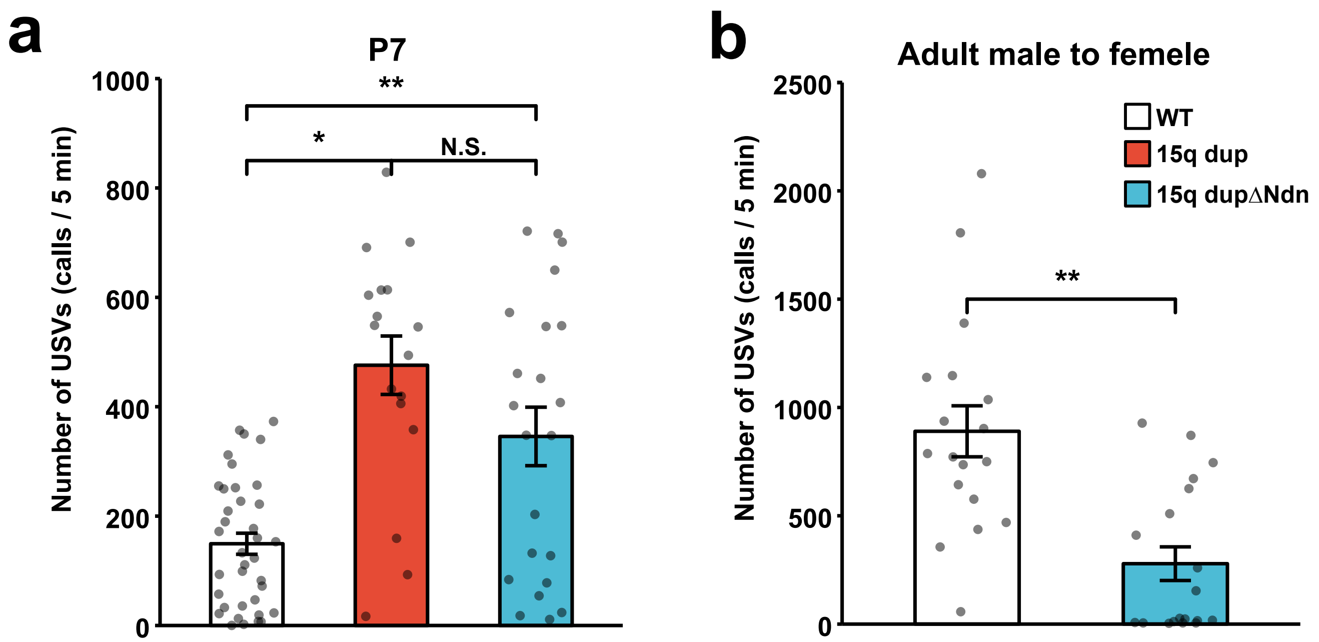

Supplementary Figure 8. Altered social communications in paternal *15q dup* mice were retained in paternal *15q dup∆Ndn* mice

(a) Maternal isolation-induced pup USV (P7). N = 37, 17, 22 for WT, *15q dup*, *15q dup∆Ndn*, respectively. Both male and female mice were used for this analysis. P7 USV: WT vs. *15q dup* : p = 0.0115, WT vs. *15q dup∆Ndn* : p < 0.0001, *15q dup* vs. *15q dup∆Ndn* : p = 0.2578. *p<0.05, **p<0.01 (Steel-Dwass test). (b) Female-induced male USV in adult mice. N = 18 and 19 for WT and *15q dup∆Ndn* male mice, respectively. Adult USV: WT vs. *15q dup∆Ndn* : p = 0.0002. **p<0.01 (Mann-Whitney U test). Data are represented as mean +/- s.e.m. N.S.: not statistically significant.

Supplementary Figue 9

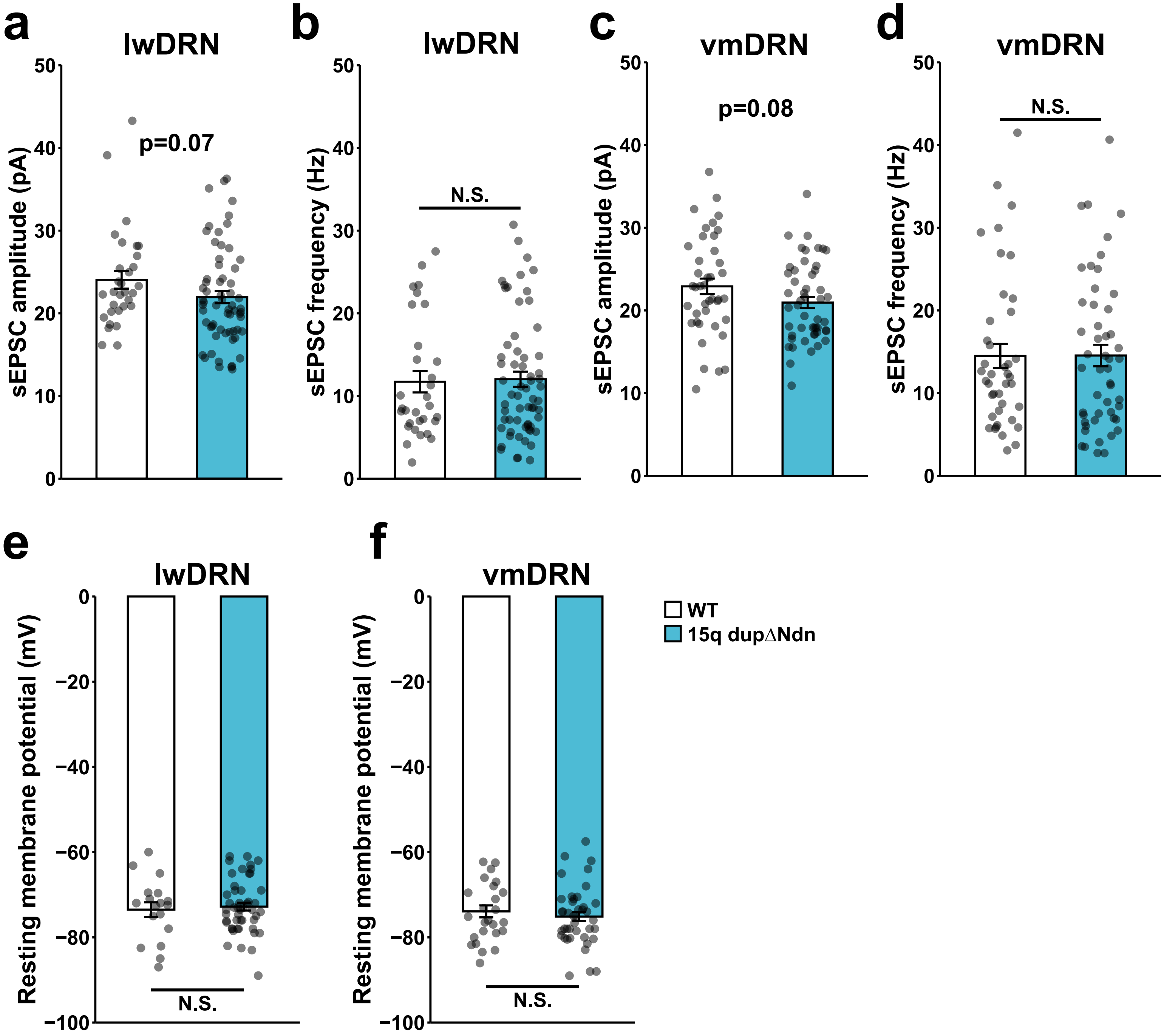

Supplementary Figure 9. Electrophysiological analyses of paternal *15q dupΔNdn* mice in the dorsal raphe nucleus

(a-d) Comparisons between WT and paternal *15q dupΔNdn* mice for sEPSC amplitude and frequency in 5-HT neurons recorded in the vmDRN and lwDRN. N = 41 (WT; vmDRN), 50 (*15q dupΔNdn*; vmDRN), 31 (WT; lwDRN), 63 (*15q dupΔNdn*; lwDRN) neurons from biologically independent 3-5 mice. **(e-f)** The resting membrane potentials of 5-HT neurons in the vmDRN and lwDRN. N = 26 (WT; vmDRN), 41 (*15q dupΔNdn*; vmDRN), 18 (WT; lwDRN), 48 (*15q dupΔNdn*; vmDRN) neurons from biologically independent 2-4 mice. Statistical testing was conducted using the Mann-Whitney U test and all parameters between genotypes did not reach the statistically significant level ($p > 0.05$). Data are represented as mean +/- s.e.m. N.S.: not statistically significant.

Supplementary Table 1 : Primer sequences

Primer name	Related figure	Sequence (5' to 3')	Used for
1.5 Mb Dp_Fw	Fig1a	AAGTAGATGTCCTAACTGACTTGC	Genotyping
1.5 Mb Dp_Rv	Fig1a	AGCTTTGAATACCATACCCAACAAA	Genotyping
Gapdh_Fw	Fig1d, 5c	ACGGGAAGCTCACTGGCATGGCCTT	qPCR
Gapdh_Rv	Fig1d, 5c	CATGAGGTCCACCACCCTGTTGCTG	qPCR
Herc2_Fw	Fig1d, 5c	TGGCCCTTTAAGCCCCAAT	qPCR
Herc2_Rv	Fig1d, 5c	GCCTATTAACCCCCAACCTATG	qPCR
Gabra5_Fw	Fig1d	TCCAAACATCCCAAAAGAGC	qPCR
Gabra5_Rv	Fig1d	GAGAGGTGGCCCCTTTTATC	qPCR
Gabrb3_Fw	Fig1d, 5c	GAATGTTGTCTTCGCCACAGGT	qPCR
Gabrb3_Rv	Fig1d, 5c	ACCCACGAGAGGATTGTGATCA	qPCR
Atp10a_Fw	Fig1d, 5c	CGCACTCACCATCTGCAATACA	qPCR
Atp10a_Rv	Fig1d, 5c	CGTGAACCTCCGAAGGAAATCT	qPCR
Ube3a_Fw	Fig1d, 5c	TCTGCTGCTGCTATGGAAGA	qPCR
Ube3a_Rv	Fig1d, 5c	CACATTCCACGTTAGGTGACA	qPCR
Ube3a_ATS_Fw	Fig1d, 5c	GGCACCCTTGTTTGAAACTT	qPCR
Ube3a_ATS_Rv	Fig1d, 5c	GCTCATGACCCTGTCCTTTC	qPCR
Snord116_Fw	Fig1d, 5c	TGGATCTATGATGATTCCCAG	qPCR
Snord116_Rv	Fig1d, 5c	TGGACCTCAGTTCCGATGAG	qPCR
Snord115_Fw	Fig1d, 5c	GGGTCAATGATGACAACCCAATG	qPCR
Snord115_Rv	Fig1d, 5c	GGGCCTCAGCGTAATCCTATTG	qPCR
Ipw_Fw	Fig1d, 5c	TCACCACAACACTGGACAAAA	qPCR
Ipw_Rv	Fig1d, 5c	TGCTGCTACACAGGAAAGAGG	qPCR
Snrpn_Fw	Fig1d, 5c	GCAAAACAGCCAGAACGTGAA	qPCR
Snrpn_Rv	Fig1d, 5c	GCACACGAGCAATGCCAGTAT	qPCR
Ndn_Fw	Fig1d, 5b, 5c	GTATCCCAAATCCACAGTGC	qPCR, ddPCR
Ndn_Rv	Fig1d, 5b, 5c	TAACTCTCCAGGGCCTTCTT	qPCR, ddPCR
Magel2_Fw	Fig1d, 5b, 5c	TTGGTGCCACTTTCTGTGCTC	qPCR, ddPCR
Magel2_Rv	Fig1d, 5b, 5c	GGCAGGAAAGGTCTCTGATGTG	qPCR, ddPCR
Mkrn3_Fw	Fig1d, 5c	AGAGCATTCTGTGCTTCGCCT	qPCR
Mkrn3_Rv	Fig1d, 5c	TACTGAAGCAAGAGCCAACGGT	qPCR
Ndn_deletion_Fw	Fig5A, FigS7a, b, e	TAGTCCCAGGTTACCAAGGC	Genotyping, deletion detection PCR
Ndn_deletion_Rv	Fig5A, FigS7a, b, e	CCTCCCACATGAGTCTGCTG	Genotyping, deletion detection PCR
Htr1a_Fw	Fig5b	CAACTATCTCATCGGCTCCT	ddPCR
Htr1a_Rv	Fig5b	GTCCTCTTGTTCACGTAGTC	ddPCR
Herc2_ddPCR_Fw	Fig5b	ATAGACACAGATGACTATGCTC	ddPCR
Herc2_ddPCR_Rv	Fig5b	GTTTCCATCTTAGTGTCCTGTC	ddPCR
Peg12_Fw	Fig5b, 5c	TTTTCCCCCTTGTTTCTGCTG	qPCR, ddPCR
Peg12_Rv	Fig5b, 5c	TGTCCCTGGAGTGCCCATTAT	qPCR, ddPCR
Cyfip1_Fw	Fig5c	CTTGACACCCAGCGAAAAAC	qPCR
Cyfip1_Rv	Fig5c	TACTCACACTCCCATCCATCAG	qPCR
Chrna7_Fw	Fig5c	CTTGATAGCACAGTACTTCG	qPCR
Chrna7_Rv	Fig5c	CCATGCACACCAATTCAG	qPCR
